# Effects of immersing treatment of curcumin and piperine combined with vacuum packaging on the quality of salmon (*Salmo salar*) during cold chain logistics

**DOI:** 10.3389/fnut.2022.1021280

**Published:** 2022-11-03

**Authors:** Yun-Fang Qian, Jia-Yi Yu, Ying-Jie Yu, Jing Xie, Sheng-Ping Yang

**Affiliations:** ^1^College of Food Science and Technology, Shanghai Ocean University, Shanghai, China; ^2^Shanghai Engineering Research Center of Aquatic Product Processing and Preservation, Shanghai Ocean University, Shanghai, China; ^3^Department of Food and Nutrition, University of Helsinki, Helsinki, Finland

**Keywords:** plant-resource preservatives, temperature abuse, temperature fluctuation, near-infrared spectroscopy, low-field nuclear magnetic resonance, free amino acid, texture profile analysis

## Abstract

In order to study the effects of the compound preservatives (curcumin and piperine (CP)) and vacuum packaging (VP) on the quality of salmon during cold chain logistics suffered from temperature abuse, the physiochemical indexes (texture, water holding capacity (WHC), total volatile basic nitrogen (TVB-N), thiobarbituric acid reactive substances (TBARS), free amino acids (FAA) contents), microbial indicators (total mesophilic bacteria count (MBC), total psychrotrophic bacteria count (PBC), H_2_S-producing bacteria count (HSBC)) were determined, and the moisture changes were explored by near-infrared (NIR) spectroscopy and low-field nuclear magnetic resonance (LF-NMR). The results showed that the treatment of curcumin and piperine in combination with vacuum packaging could maintain the quality of salmon suffered from temperature abuse most effectively. At the end of storage, the MBC of VP+CP was only 4.95 log CFU/g, which was about 1 log CFU/g lower than the control sample stored at the same condition. The combined treatment also retarded the increase of TVB-N, TBARS, and the decrease of hardness, springiness, and a* value, as well as water migration in salmon, contributing to higher water holding capacity and better appearance. Besides, VP+CP retarded the decrease of free glutamate, which contributed to umami taste. Due to the biological activity and safety of the preserves, the combined treatment could be a promising method for preservation of seafood.

## Introduction

Natural extracts from plant attract much interest from scientists and consumers for their various biological activities, such as anticancer, anti-obesity, antispasmodic effects, etc. (1, 2). The use of these extracts for food preservation is also a trend followed by both consumers and food manufacturers as a replacement of synthetic preservatives regarding to the health concern (3). Compared to synthetic chemicals, natural preservatives are Generally Recognized as Safe (GRAS) (4–6). The present knowledge shows that the plant extracts display antibacterial, antiviral, antifungal, and antioxidant effects, which can strongly reduce pathogenic and spoilage microorganisms in foods (7–9). However, these extracts can be very complex, which are composed of at least 50 components, making it difficult to predict the susceptibility of a microorganism to a kind of plant extracts (10). Therefore, it is necessary to study the effect of the extracts on different food products individually. Curcumin is a natural pigment mainly extracted from the rhizomes or tubers of various ginger plants, which has been widely used in the medical field because of its anti-inflammatory properties (11), promoting wound healing (12), regulating protein and metabolic pathways (13), inhibiting tumor growth and other pharmacological effects (14). Curcumin is also a food additive known for its antibacterial and antioxidant activity (15). In recent years, as a natural and effective biological preservative, the effect of curcumin on the preservation of many raw and processed food products, such as sturgeon (16), surimi (17), millet fresh noodles (18), and kiwi fruit (19) has been proven. Piperine is another bioactive ingredient extracted from pepper, which possesses hot and pungent characters (20). This compound has attracted the attention of scientists due to its numerous benefits including its antioxidant activity, anti-inflammatory activity, and bio-enhancing ability (21, 22). It not only has therapeutic effects but also can be used as a functional ingredient in food (23, 24). Studies have shown that the use of composite preservatives on aquatic products is better than that of single preservative, mainly due to the enhanced synergistic effect (25).

Salmon (*Salmo salar*) is favored by consumers for its nutritional value and culinary properties, whose global market size is growing constantly (26). At present, salmon is mainly produced in Norway, followed by Chile, UK, North America, the Faroe Island, etc. Since salmon is rich in moisture, protein, and polyunsaturated fatty acids, it is highly perishable during storage, transportation, and sales processes with a shelf life as short as several days even at chilling environment (27, 28). To make things worse, it is very difficult to keep the temperature stable during cold chain logistics, especially when the cargos are exposed to outdoors in the loading and uploading process (29). In recent years, the effect of temperature abuse during cold chain logistic has been concerned by researches (30–32).

In our previous study, the effectiveness of curcumin and piperine on inhibiting bacteria and prolonging the shelf life of salmon were illustrated and the optimal formula was settled (33). However, the preservatives seem to have a much greater sensitivity to Gram-positive than Gram-negative bacteria (34), and the dominant spoilers of salmon are Gram-negative bacteria (35). Therefore, to enhance the effect of curcumin and piperine, a combined technique should be employed.

Packaging is a commonly applied technology to maintain the quality of aquatic products during refrigeration. Studies have shown that the combination of vacuum packaging and low-temperature refrigeration can inhibit the growth of aerobic microorganisms (36), so that the proportion of anaerobic bacteria such as lactic acid bacteria can increase (37). Therefore, it is hypothesized that the vacuum packaging may have a synergistic effect with curcumin and piperine. To our knowledge, little research has illustrated the efficacy of vacuum packaging in combination with curcumin and piperine at unsuitable temperatures that can be common during the whole distribution period. Based on this, the objective of this study is to determine the changes of microbial growth, physiochemical indicators, texture properties and water distribution of salmon suffered from temperature abuse, and provide a potential method to prolong the shelf life of salmon.

## Materials and methods

### Materials and reagents

Plate count agar (PCA) and iron agar medium were purchased from Qingdao Hope Bio-Technology Co., Ltd. (Qingdao, Shandong, China). Magnesium oxide, boric acid, sodium chloride, and 2-thiobarbituric acid were purchased from Sangon Biotech (Shanghai) Co., Ltd. (Shanghai, China). Other agents of analytic grade were purchased from Shanghai Aladdin Biochemical Technology Co., Ltd. (Shanghai, China).

### Samples preparation

**FIGURE 1:**
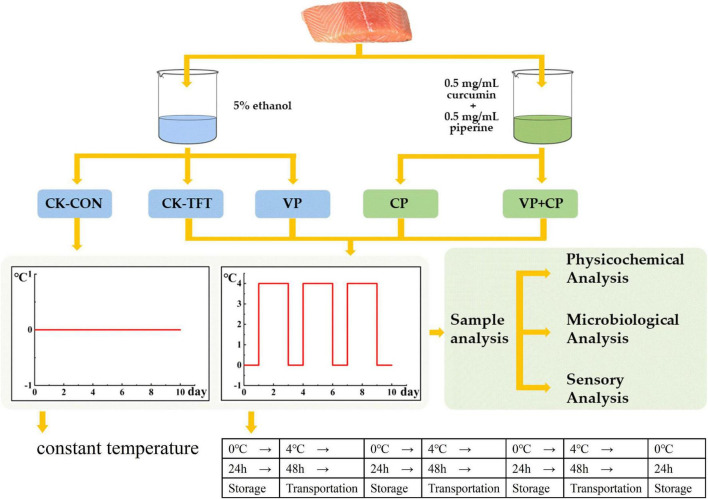
A schematic overview of the designation and process of this study.

The chilled salmon filets were purchased from local market (Pudong District, Shanghai, China) and transported to the laboratory within 30 min in an incubator with some ice chips (about 0∼4°C). The salmon were divided equally and randomly into five groups (18 pieces for each treatment, about 120 g each piece). Each group was marked and treated according to the schematic overview of the key stages of the experimental study (Figure 1). For the preservative treatment (CP), a combined solution (5% ethanol) composed of 0.5 mg/mL curcumin and 0.5 mg/mL piperine was prepared beforehand according to the previous study (33). The treatments and storage conditions for each group were demonstrated as follows:

(1) CK-TFT: The salmon was soaked in 5% ethanol solution for 30 s and then stored under fluctuation conditions of 0 °C and 4°C to simulate the temperature fluctuation during cold chain logistics.
(2) CK-CON: The salmon was soaked in 5% ethanol solution for 30 s and then stored at a constant temperature (0°C).
(3) VP: The salmon was soaked in 5% ethanol solution for 30 s, then vacuum-packed, and stored under temperature fluctuation conditions.
(4) CP: The salmon was soaked in 0.5 mg/mL curcumin + 0.5 mg/mL piperine compound solution for 30 s and then stored under temperature fluctuation conditions.
(5) VP+CP: The salmon was soaked in 0.5 mg/mL curcumin + 0.5 mg/mL piperine compound solution for 30 s, then vacuum-packed and stored under temperature fluctuation conditions.

During the 10-day experimental period, the sampling was carried out every two days.

### Microbial analysis

The total mesophilic bacterial counts (MBC), total psychrotrophic bacteria count (PBC), and H_2_S-producing bacterial counts (HSPBC) of salmon filets were determined according to Yu et al. (28). In brief, 25 g meat sample were taken from each group under sterile condition and homogenized with 225 mL of 0.85% sterilized saline water with a stomacher blender for 1 min. From this dilution, a series of dilutions (1:10) were prepared in 0.1% peptone, and 0.1 mL of each dilution was dispensed onto the appropriate medium. Plate count agar (Qingdao Hope Bio-Technology Co., Ltd., Shandong, China) was used for determining total mesophilic bacteria count (MBC) (48 h incubation at 30 °C) and total psychrotrophic bacteria count (PBC) (10 d incubation at 4 °C). The H_2_S-producing bacteria count was determined by counting black colonies on Iron agar (Qingdao Hope Bio-Technology Co., Ltd., Shandong, China) after incubation at 25°C for 72 h. Each dilution was performed in three parallels.

### Determination of total volatile base nitrogen

Total volatile base nitrogen (TVB-N) content was determined using a Kjeldahl automatic distillation apparatus (Kjeltec™ 8400, FOSS, Denmark) according to the method reported by Holman et al. (38). The TVB-N value is calculated according to the consumption of hydrochloric acid (0.1 mol/L) and expressed in mg/100g fish sample. The experiments were carried out in triplicate.

### Determination of water-holding capacity

The water-holding capacity of fish meat was carried out according to the method of Gudjónsdóttir et al. (39) with some modifications. Briefly, fish meat (approximately 2 g, M1) was accurately weighed and the sample was accurately weighed again (M2) after centrifugation at 5,000 *g* for 10 min at 4°C. The experiments were carried out in triplicate. The WHC was calculated according to the following formula:


(1) $$\mathrm{WHC}\%=\left(1-\frac{\text{M}\,1-\text{M}\,2}{\text{M}\,1}\right)\times 100\%$$

### Determination of thiobarbituric acid reactive substances

Thiobarbituric acid reactive substances (TBARS) content was measured according to the method reported by Wang et al. (40). In brief, the salmon meat (5 g) was homogenized with 25 mL of 20% (w/v) trichloroacetic acid (Sinopharm Chemical Reagent Co., Ltd., Shanghai, PR China). After incubation at 0°C for 1 h, the mixture was centrifuged at 8,000 *g* at 4°C for 10 min. The supernatant was mixed with 0.02 M 2-thiobarbituric acid and heated at 100°C for 20 min. Then, the absorbance was measured at 532 nm, and the results were expressed as mg malondialdehyde per kg fish sample (mg/kg). The experiment was conducted in triplicate.

### Texture profile analysis

The hardness and springiness were performed using a TA.XT Plus texture analyzer (Stable Micro Systems Ltd., Surrey, UK). The fish samples were quickly cut into 10 mm × 10 mm × 10 mm squares at a constant temperature of 20°C. The test conditions were settled according to Wang, Xiang, Fan, Xie, Qian (40). The sample was compressed using a cylindrical probe of 6 mm in diameter (P/6) on the platform under the following conditions: pre-test speed, 3 mm/s; constant test speed, 1 mm/s; post-test speed, 1 mm/s, sample deformation, 50%. Six measurements for each group were carried out.

### Colorimetric measurement

The L*, a*, and b* values of salmon samples were measured by a CR-400 type colorimeter (Konica Minolta Investment Ltd., Tokyo, Japan) according to the protocols, and six parallel experiments were performed for each group.

### Determination of free amino acids

The contents of free amino acids was determined using the method of Tavakoli et al. (41) with slightly modifications. Briefly, a minced sample (1.0 g) was homogenized with 10 mL of 5% trichloroacetic acid and centrifuged at 8,000 *g* for 10 min at 4°C. The precipitate was centrifuged again. The two supernatants were combined and diluted to 25 mL with deionized water. A 1 mL extract was obtained by filtration using a disposable filter and analyzed by a LA-8080 ultra-high-speed amino acid automatic analyzer (©Hitachi, Ltd., Tokyo, Japan). The experiment was performed in triplicate.

### Near-infrared spectroscopic analysis

Each fillet (2 cm × 2 cm × 2 cm) was measured by an NIR instrument (NIRFlex Solids N-500 spectrophotometer, Büchi Labor-technik AG, Flawil, Switzerland) in reflectance mode. The spectra from 4,000 to 10,000 cm^–1^ were recorded and digitalized with ca. 8 cm^–1^ intervals in the Fourier transform. Both air reference and the sample spectra were measured with scan number 64. The temperature range was 20 to 25°C. The experiment was conducted in triplicate and the average spectrum of each sample was recorded.

### Low-field nuclear magnetic resonance

The measurements were determined using an low-field nuclear magnetic resonance (LF-NMR) analyzer (PQ 001, Shanghai Niumag Electronic Technology Co., Ltd., Shanghai, China) following the method reported by Yu et al. (28). The transverse relaxation time of each fillet (2 cm × 2 cm × 2 cm) was determined using the Carr-Purcell-Meiboom-Gill sequence at 32°C. The parameters applied were proton resonance frequency = 24 MHz, sampling interval time (TW) = 2,000 ms, echo number (NECH) = 8,000, Pre-amplifier gain (PGR) = 1, cumulative number (NS) = 4, echo time = 0.5 ms, pulse width at 90° (P1) = 18.00 μs, and pulse width at 180° (P2) = 34.00 μs. The signals were collected to obtain the transverse relaxation time T_2_ spectrum by software (NMI20-030H-1 NMR analyzer: Suzhou Niumag Analytical Instrument Co., Suzhou, China). Magnetic resonance imaging (MRI) was performed with the same LF-NMR analyzer and a spin-echo sequence, and the gray-scale map of the proton density distribution was obtained. The gray-scale image was altered to be pseudocolor images by MATLAB software (MathWorks Inc., Natick, MA, USA).

### Statistical analysis

All samples were conducted at least in triplicate. To investigate the difference among different variables, an ANOVA (one-way analysis of variance) followed by the least significant difference (LSD) test were performed by SPSS 20.0 (SPSS Inc., Chicago, IL, USA) (*P* < 0.05). The correlation analysis was carried out using Pearson’s method, using the listwise method to exclude missing values. It assigns a value between −1 and 1, where 0 is no correlation, 1 is total positive correlation, and −1 is total negative correlation. Differences were considered significant if *P* < 0.05. Diagrams were performed by Origin 2021 (OriginLab Corporation, Northampton, MA, USA).

## Results and discussion

### Changes of microbial counts

**FIGURE 2:**
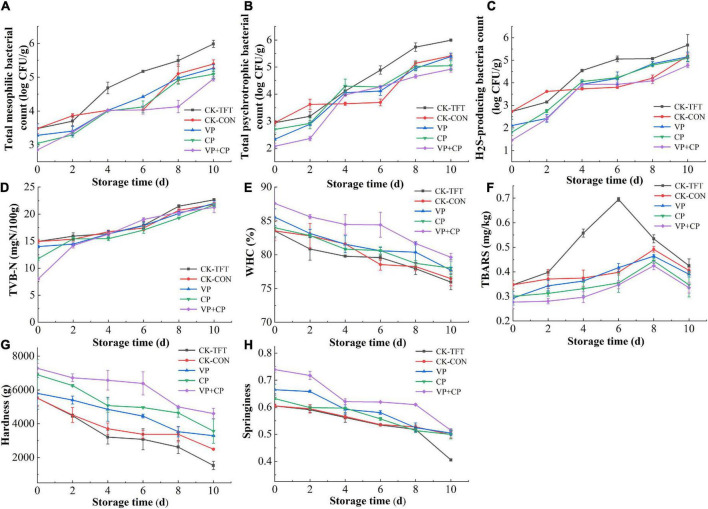
Changes of microbiological and physiochemical indicators of salmon treated by curcumin and piperine combined with vacuum packaging during cold chain logistics: **(A)** Total mesophilic bacteria count (MBC); **(B)** total psychrotrophic bacteria count (PBC); **(C)** H_2_S-producing bacteria count (HSPBC); **(D)** TVB-N; **(E)** WHC; **(F)** TBARS; **(G)** hardness; **(H)** springiness.

The changes in MBC of the salmon filets are presented in Figure 2A. The initial TVC of the untreated fresh salmon samples was 3.48 log CFU/g, indicating the fillet was in a fresh state. The MBCs of the fresh salmon samples treated with different vacuum-packaging and preservatives were slightly lower than the untreated sample, i.e., 3.28 log CFU/g, 3.04 log CFU/g, and 2.85 log CFU/g on day 0, respectively. It indicated that the VP and CP treatment could inhibit the bacteria immediately after treatment, and CP was more effective than VP. With the increase storage time, the MBC in all groups increased with varied rates, but none of the groups exceeded the maximum acceptable limit (>7 log CFU/g) during the whole storage period (42). The MBC and PBC in CK-TFT were significantly higher than those in the other four groups, indicating that temperature fluctuation could promote the proliferation of bacteria (28). At the end of storage, the MBC of the CK-TFT reached 5.98 log CFU/g, while VP+CP was only 4.95 log CFU/g, which was even lower than CK-CON sample stored at constant 0°C. Therefore, the combined treatment with preservatives and vacuum packaging could inhibit the growth of bacteria most effectively. It is reported that curcumin and piperine display a higher inhibitory effect against Gram-positive bacteria than Gram-negative bacteria (34), while vacuum packaging was favored by lactic acid bacteria, which are Gram-positive bacteria (37, 43). The result confirmed our hypothesis that curcumin and piperine could complement the inhibition effect of vacuum packaging.

The growth trend of PBC was similar to the change of MBC (Figure 2B). At the end of storage, PBC in groups CK-TFT and CK-CON reached 5.99 log CFU/g and 5.41 log CFU/g, and PBC in treatment groups was 5.39 log CFU/g, 5.05 log CFU/g and 4.92 log CFU/g, respectively. This showed that psychrotrophic bacteria were predominant bacteria of salmon during cold chain logistics. The curcumin and piperine combined with vacuum packaging could effectively inhibit the growth and reproduction of microorganisms, followed by preservative treatment and vacuum treatment alone. The antibacterial effect of curcumin and piperine was confirmed by the results in this study. It is demonstrated that curcumin and piperine can inhibit bacteria by targeting the bacterial cell membrane, cell wall, protein, DNA, and/or by inhibiting bacterial growth through the quorum sensing (QS) system (44, 45).

H_2_S-producing bacteria are one of the most important spoilage bacteria in aquatic products, mainly *Shewanella* species, which can produce trimethylamine (TMA), H_2_S, and biogenic amines (46). As shown in Figure 2C, the H_2_S-producing bacteria count in the five groups increased with the increasing storage time, but the value was lower than MBC. The initial H_2_S-producing bacteria count in the fresh salmon samples of the CK groups was 2.72 log CFU/g. At the end of storage, the H_2_S-producing bacteria count in groups CK-TFT and CK-CON reached 5.67 log CFU/g and 5.20 log CFU/g, while the treatment groups were only 5.15 log CFU/g, 5.09 log CFU/g, and 4.77 log CFU/g, respectively. It was found that both preservative treatment and vacuum packaging could reduce the HSPBC to a certain extent, and the combined treatment showed the highest effectiveness.

### Changes of total volatile base nitrogen

Total volatile base nitrogen is one of the most important indicators for the freshness of seafood, which is produced by endogenous and bacterial enzymes (47, 48). As shown in Figure 2D, the TVB-N of the five groups of samples under different storage conditions gradually increased with the extension of storage time. The initial TVB-N of the salmon samples in the control group was 14.96 mg N/100g, indicating that the salmon samples were fresh. During storage, the TVB-N values of all groups increased continually. At the later stage of storage, the TVB-N of VP+CP was lower than the control groups, indicating that vacuum packaging and preservative treatment could reduce the formation of spoilage products and delay the spoilage of salmon. The inhibition effect of the treatments on TVB-N should be contributed to the antibacterial activity of vacuum packaging and the preservatives.

### Changes of water holding capacity

Water holding capacity can be used to reflect the ability of aquatic products to retain water during storage (49). The WHC of salmon samples in the cold chain logistics process under different treatment conditions is shown in Figure 2E. During storage, the WHC of each treatment group showed a downward trend, but the WHC of the treatment groups was higher than that of CK-TFT, especially in VP+CP. The WHC of fresh salmon after compound preservative combined with vacuum packaging was 87.58% on day 0, and decreased to 79.62% at the end of storage. The initial WHC of the CK-TFT and CK-CON was 83.52% on day 0, but decreased to 75.94% and 76.45% on day 10, respectively. It is hypothesized that vacuum packaging helped the fish muscle to retain more moisture due to the compression of the normal atmosphere after immersing treatment of preservative solution. During the whole period, the WHC of the combined treatment was also obviously higher than all other four groups, indicating that VP+CP could conquer the negative effect of temperature abuse, and had a synergetic effect on retaining moisture in comparison with VP or CP alone. CP also had higher WHC than CK-CON and CK-TFT, which could be contributed to its action against muscle denaturation and myofibril structures destruction (50).

### Changes of thiobarbituric acid reactive substances

Salmon is rich in unsaturated fatty acids, which degrade to volatile compounds including aldehydes, ketone, alcohols, etc., leading to off-odors and color changes (51, 52). The secondary oxidation product of the lipids was determined by TBARS (53), and the results are displayed in Figure 2F. The TBARS of all groups increased in the early stage and then decreased after the peak. The TBARS of CK-TFT increased more rapidly than other groups, and reached the peak (0.69 mg/100g) on day 6, while the other four groups reached the lower maximum values on day 8. The TBARS values of VP, CP, and VP+CP were even lower than CK-CON, which indicated that the treatments could inhibit the lipid oxidation effectively and eliminate the adverse effect of temperature abuse. The polyphenols in CP and limited oxygen in VP should contribute to this phenomenon (21, 54). The decrease of TBARS after the peak in all groups might be due to the further degradation of malondialdehyde.

. With the increase of storage time, drip loss can be explained by the water loss in muscle, which may be due to the gradual increase in the water-holding properties of muscle proteins with the loss of protein degradation, which is manifested in the decrease of muscle hardness and springiness.

### Changes of hardness and springiness

During the cold chain logistics process, the hardness and springiness of salmon decreased with the increasing time (Figures 2G,H). The initial hardness and springiness of the fresh samples increased to a certain extent after treatment, which might be due to the increase of moisture in the muscle after immersing treatment, and slight pressure by normal atmosphere after vacuum packaging. During simulated cold chain logistic, the hardness and springiness decreased due to the proteolytic degradation of protein and drip loss (55). Among the treatment groups, the hardness and springiness of VP+CP were maintained at a highest level, followed by CP and VP, indicating that the vacuum packaging combined with the compound preservatives could retard the degradation of protein most effectively.

### Changes of colorimetric properties

Color is an important factor affecting consumer acceptance of aquatic products (56). The L*, a*, and b* values of salmon in the process of simulating cold chain logistics are shown in Table 1. After treatment by CP, the b* value increased slightly in comparison with CK on day 0, which might be due to curcumin. But the differences were not significant (P > 0.05). Therefore, the treatment did not bring adverse effect on appearance. The L* (lightness) values of all groups were fluctuating upward, and VP+CP was generally lower than other groups. The changes in L* values can be contributed to the oxidation and denaturation of proteins (57). Thus, VP+CP might inhibit the denaturation and oxidation of proteins due to the antioxidant compounds and anaerobic atmosphere.

**TABLE 1:** Effects of different treatments on L*, a*, and b* of salmon in cold chain logistics.

		0d	2d	4d	6d	8d	10d
L*	CK-TFT	47.27 ± 2.56^a^	48.93 ± 2.79^a^	50.03 ± 1.57^b^	50.24 ± 4.58^ab^	45.62 ± 2.18^a^	51.43 ± 0.19^ab^
	CK-CON	47.27 ± 2.56^a^	46.07 ± 3.04^ab^	49.92 ± 1.22^b^	43.87 ± 7.48^bc^	49.00 ± 3.94^a^	54.49 ± 5.38^a^
	VP	40.69 ± 1.57^b^	44.25 ± 2.02^b^	45.11 ± 2.37^c^	39.78 ± 5.33^c^	45.62 ± 1.25^a^	53.84 ± 4.29^a^
	CP	49.70 ± 2.92^a^	47.81 ± 2.30a^b^	60.91 ± 0.92^a^	57.12 ± 2.99^a^	46.10 ± 4.08^a^	51.17 ± 2.51^ab^
	VP+CP	48.41 ± 2.18^a^	49.66 ± 1.90^a^	49.41 ± 3.20^b^	54.17 ± 5.19^a^	45.45 ± 1.73^a^	47.85 ± 2.77^b^
a*	CK-TFT	11.60 ± 0.89^a^	11.66 ± 0.81^a^	11.73 ± 1.70^a^	10.49 ± 0.39^a^	12.06 ± 1.40^a^	10.79 ± 0.36^a^
	CK-CON	11.60 ± 0.89^a^	10.20 ± 0.72^a^	11.56 ± 1.20^a^	9.88 ± 0.79^a^	10.79 ± 1.59^a^	10.87 ± 1.60^a^
	VP	9.81 ± 0.72^a^	8.24 ± 1.29^b^	8.97 ± 0.59^b^	8.34 ± 0.56^b^	11.43 ± 0.69^a^	11.80 ± 1.54^a^
	CP	11.49 ± 1.74^a^	10.15 ± 1.42^a^	10.62 ± 1.03^ab^	9.64 ± 0.88^a^	11.75 ± 1.81^a^	11.44 ± 1.65^a^
	VP+CP	11.31 ± 1.69^a^	10.70 ± 0.39^a^	11.36 ± 0.64^a^	10.48 ± 0.90^a^	11.37 ± 0.83^a^	11.69 ± 0.47^a^
b*	CK-TFT	19.53 ± 1.64^ab^	21.90 ± 2.46^ab^	21.86 ± 4.12^a^	19.79 ± 1.52^a^	21.28 ± 1.74^a^	19.38 ± 2.32^a^
	CK-CON	19.53 ± 1.64^ab^	17.75 ± 0.94^c^	20.55 ± 2.47^a^	20.12 ± 2.10^a^	20.17 ± 3.41^a^	21.66 ± 3.46^a^
	VP	16.91 ± 0.86^b^	18.71 ± 3.09^bc^	19.60 ± 1.41^a^	15.06 ± 0.79^b^	20.35 ± 2.08^a^	21.53 ± 1.05^a^
	CP	21.46 ± 2.84^a^	22.32 ± 1.88^a^	20.13 ± 1.97^a^	18.39 ± 3.47^a^	23.08 ± 2.74^a^	21.98 ± 4.14^a^
	VP+CP	21.85 ± 2.18^a^	21.17 ± 2.13^abc^	24.04 ± 2.71^a^	20.22 ± 2.24^a^	25.06 ± 6.13^a^	22.27 ± 3.25^a^

Different lowercase letters indicate significant differences (*P* < 0.05) among different treatments within the same storage time.

The changes of a* value (redness) during the whole period is shown in Table 1. Salmon fillet displays typical orange-red color due to the richness of astaxanthin, which is an antioxidant. The a* value of CK-TFT and CK-CON slightly decreased due to the oxidation process (58). The results displayed that the treatments of VP, CP, and VP+CP could maintain the a* value by preventing the oxidation of astaxanthin (59).

In this study, the value of b* (yellowness) in all treatment groups increased slightly during the storage (Table 1), but there were no significant differences between groups (*P* > 0.05). An increase of b* values of catfish during storage were also reported by Peterman et al. (60).

### Changes of free amino acids concentrations

Free amino acids not only provide the umami, bitterness, and sweetness of aquatic foods, and they are also precursors of biogenic amines, ammonia, methanthiol, and other toxic or off-odor compounds by bacteria and endogenous enzymes (61). The changes of FAA contents in salmon during the cold chain logistics are shown in Table 2. The changes of FAAs contents are very complicated, which depends on the balance between the proteolytic hydrolysis rate from protein or peptide and their degradation rate by bacteria and endogenous enzymes.

**TABLE 2:** Effects of different treatments on free amino acid content (mg/100 g) of salmon in cold chain logistics.

Storage time	Treatment	Asp	Thr	Ser	Glu	Gly	Ala	Cys	Val	Met
0d	CK	0.49 ± 0.00^b^	9.35 ± 0.01^b^	4.51 ± 0.02^a^	22.77 ± 0.10^c^	12.40 ± 0.06^b^	22.98 ± 0.64^d^	1.02 ± 0.01^c^	6.34 ± 0.08^c^	2.47 ± 0.26^ab^
	VP	0.30 ± 0.01^d^	8.37 ± 0.04^c^	4.05 ± 0.04^b^	24.30 ± 0.09^b^	13.09 ± 0.10^a^	28.24 ± 0.14^a^	1.27 ± 0.03^a^	6.93 ± 0.07^b^	2.15 ± 0.03^b^
	CP	0.37 ± 0.01^c^	7.61 ± 0.08^d^	3.75 ± 0.02^c^	26.12 ± 0.16^a^	10.18 ± 0.01^c^	25.57 ± 0.12^b^	1.03 ± 0.01^c^	7.02 ± 0.05^ab^	2.64 ± 0.13^a^
	VP+CP	0.74 ± 0.01^a^	12.43 ± 0.03^a^	4.51 ± 0.01^a^	22.01 ± 0.09^d^	13.20 ± 0.03^a^	23.61 ± 0.06^c^	1.02 ± 0.03^b^	7.13 ± 0.01^a^	2.42 ± 0.11^ab^
4d	CK-TFT	1.00 ± 0.03^c^	8.44 ± 0.04^b^	4.90 ± 0.02^c^	21.14 ± 0.19^d^	10.22 ± 0.08^e^	27.63 ± 0.20^d^	1.05 ± 0.01^c^	8.29 ± 0.26^b^	3.10 ± 0.34^b^
	CK-CON	0.28 ± 0.01^d^	7.97 ± 0.09^d^	3.57 ± 0.02^d^	20.87 ± 0.05^d^	11.38 ± 0.03^d^	26.00 ± 0.06^e^	1.06 ± 0.01^c^	7.88 ± 0.39^b^	2.39 ± 0.28^c^
	VP	1.76 ± 0.01^b^	9.34 ± 0.04^a^	4.98 ± 0.02^c^	25.33 ± 0.20^b^	12.21 ± 0.05^c^	30.65 ± 0.07^c^	1.21 ± 0.02^b^	9.63 ± 0.02^a^	3.70 ± 0.07^a^
	CP	0.12 ± 0.01^e^	6.24 ± 0.16^e^	5.91 ± 0.15^b^	24.02 ± 0.12^c^	15.55 ± 0.03^a^	35.66 ± 0.11^a^	1.38 ± 0.04^a^	8.18 ± 0.09^b^	2.91 ± 0.12^b^
	VP+CP	1.81 ± 0.00^a^	8.15 ± 0.04^c^	6.25 ± 0.08^a^	26.67 ± 0.24^a^	12.93 ± 0.12^b^	31.87 ± 0.06^b^	1.22 ± 0.03^b^	9.87 ± 0.26^a^	3.98 ± 0.35^a^
10d	CK-TFT	0.43 ± 0.01^c^	8.27 ± 0.00^d^	6.60 ± 0.02^b^	14.05 ± 0.04^c^	13.56 ± 0.01^e^	40.03 ± 0.06^b^	1.08 ± 0.01^c^	11.76 ± 0.04^a^	4.70 ± 0.02^b^
	CK-CON	0.35 ± 0.00^d^	8.64 ± 0.06^c^	5.12 ± 0.07^c^	11.21 ± 0.20^e^	15.29 ± 0.14^c^	39.98 ± 0.15^b^	1.43 ± 0.02^ab^	10.04 ± 0.10^d^	3.45 ± 0.40^d^
	VP	2.15 ± 0.04^a^	10.81 ± 0.12^a^	6.95 ± 0.07^a^	19.77 ± 0.20^b^	15.51 ± 0.15^b^	40.99 ± 0.41^a^	1.44 ± 0.03^a^	11.60 ± 0.12^b^	5.44 ± 0.31^a^
	CP	0.30 ± 0.01^e^	10.08 ± 0.02^b^	3.61 ± 0.05^d^	12.37 ± 0.19^d^	16.71 ± 0.15^a^	40.88 ± 0.15^a^	1.47 ± 0.06^a^	10.19 ± 0.05^d^	4.04 ± 0.20^c^
	VP+CP	0.58 ± 0.02^b^	5.98 ± 0.09^e^	6.98 ± 0.04^a^	20.83 ± 0.21^a^	14.84 ± 0.02^d^	37.38 ± 0.10^c^	1.38 ± 0.00^b^	10.37 ± 0.10^c^	3.54 ± 0.40^cd^

**Storage time**	**Treatment**	**Ile**	**Leu**	**Tyr**	**Phe**	**Lys**	**His**	**Arg**	**Pro**	**Total amino acid content**

0d	CK	2.43 ± 0.31^a^	4.76 ± 0.20^b^	4.72 ± 0.13^c^	3.02 ± 0.15^b^	112.70 ± 1.16^c^	17.00 ± 0.17^d^	3.86 ± 0.05^b^	1.77 ± 0.09^b^	232.58 ± 2.66^c^
	VP	2.58 ± 0.04^a^	5.08 ± 0.09^b^	5.04 ± 0.11^b^	2.62 ± 0.09^b^	167.05 ± 4.75^a^	17.56 ± 0.42^c^	1.93 ± 0.03^c^	1.17 ± 0.12^c^	291.76 ± 5.81^ab^
	CP	2.89 ± 0.34^a^	5.59 ± 0.31^a^	5.94 ± 0.29^a^	4.01 ± 0.50^a^	163.14 ± 2.05^a^	25.73 ± 0.30^b^	1.90 ± 0.02^c^	1.19 ± 0.21^c^	294.69 ± 3.82^a^
	VP+CP	2.55 ± 0.01^a^	5.88 ± 0.02^a^	5.79 ± 0.01^a^	4.30 ± 0.04^a^	143.38 ± 1.40^b^	30.72 ± 0.22^a^	4.63 ± 0.06^a^	2.04 ± 0.10^a^	286.43 ± 1.91^b^
4d	CK-TFT	2.96 ± 0.01^c^	8.22 ± 0.07^c^	7.69 ± 0.17^c^	7.48 ± 0.05^c^	159.90 ± 0.43^c^	25.03 ± 0.08^c^	2.88 ± 0.03^c^	1.81 ± 0.12^ab^	301.77 ± 0.49^c^
	CK-CON	2.70 ± 0.02^d^	6.67 ± 0.04^d^	6.78 ± 0.01^d^	5.57 ± 0.02^d^	129.65 ± 0.36^e^	24.41 ± 0.11^d^	2.53 ± 0.01^d^	1.36 ± 0.58^bc^	261.09 ± 0.50^e^
	VP	3.49 ± 0.12^b^	9.90 ± 0.06^b^	9.11 ± 0.03^b^	8.67 ± 0.03^b^	185.72 ± 0.97^b^	31.18 ± 0.15^b^	3.09 ± 0.01^b^	1.90 ± 0.04^ab^	351.86 ± 0.97^b^
	CP	2.59 ± 0.17^d^	5.77 ± 0.13^e^	5.38 ± 0.10^e^	3.55 ± 0.08^e^	151.48 ± 1.55^d^	19.48 ± 0.16^e^	1.89 ± 0.03^e^	0.90 ± 0.29^c^	290.99 ± 2.34^d^
	VP+CP	3.69 ± 0.03^a^	10.42 ± 0.09^a^	9.64 ± 0.09^a^	9.19 ± 0.27^a^	198.46 ± 1.42^a^	32.13 ± 0.10^a^	3.54 ± 0.01^a^	2.25 ± 0.26^a^	372.07 ± 0.97^a^
10d	CK-TFT	3.56 ± 0.01^a^	11.05 ± 0.03^a^	3.69 ± 0.03^c^	10.40 ± 0.07^a^	143.91 ± 1.09^b^	11.82 ± 0.03^c^	1.49 ± 0.04^c^	2.20 ± 0.21^a^	288.59 ± 1.50^b^
	CK-CON	2.59 ± 0.01^c^	8.06 ± 0.04^d^	3.16 ± 0.02^d^	8.59 ± 0.04^c^	145.43 ± 1.22^b^	8.46 ± 0.05^e^	0.97 ± 0.02^e^	1.93 ± 0.09^b^	274.70 ± 2.30^d^
	VP	3.62 ± 0.20^a^	10.91 ± 0.20^a^	5.57 ± 0.14^a^	10.40 ± 0.19^a^	162.00 ± 1.73^a^	13.12 ± 0.14^a^	2.12 ± 0.03^a^	2.32 ± 0.03^a^	324.66 ± 3.83^a^
	CP	2.70 ± 0.24^bc^	8.51 ± 0.19^c^	3.28 ± 0.06^d^	8.59 ± 0.16^b^	143.92 ± 0.68^b^	9.16 ± 0.04^d^	1.26 ± 0.02^d^	1.93 ± 0.12^b^	279.63 ± 2.06^c^
	VP+CP	2.91 ± 0.02^b^	9.24 ± 0.06^b^	4.31 ± 0.03^b^	10.40 ± 0.09^b^	144.57 ± 1.23^b^	12.93 ± 0.09^b^	1.88 ± 0.05^b^	1.10 ± 0.12^c^	287.80 ± 2.21^b^

Different lowercase letters indicate significant differences (*P* < 0.05) among different treatments within the same storage time.

The total FAAs contents of VP, CP, and VP+CP were higher than CK on day 0. During the simulated logistics, the total FAAs contents increased first and then decreased slightly. Among them, VP had the highest level of total FAAs contents. It showed that lysine was the most abundant FAA in salmon, followed by alanine and glutamate. The content of lysine in all groups increased first and then decreased, and its decrease could be contributed to the formation of cadaverine by decarboxylase activity from bacteria (62). On day 10, VP had the highest content of lysine, and the differences between the other groups were not significant (*P* > 0.05). Glutamate can provide umami taste, which is also one of the precursors of putrescine (63). The contents of glutamate in all groups decreased during the period, but VP+CP had the highest content, indicating that VP+CP could inhibit the degradation of glutamate and maintain the umami taste most effectively.

### Near-infrared spectroscopic analysis

**FIGURE 3:**
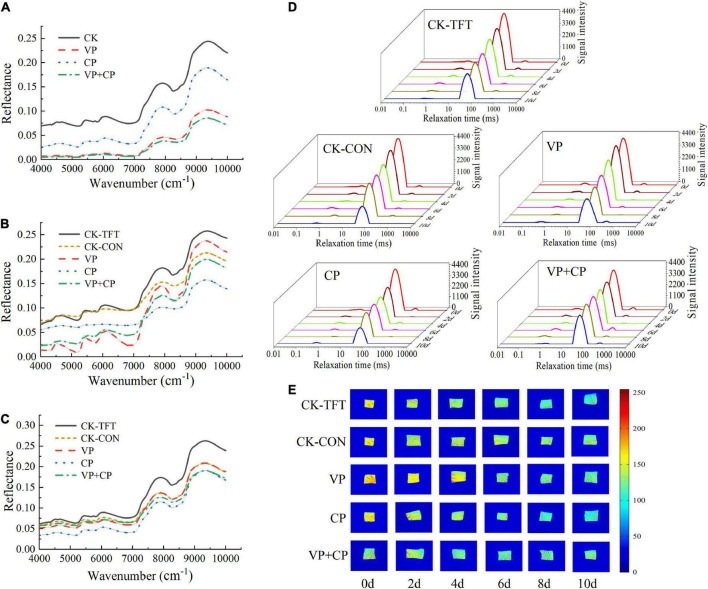
Original reflectance spectra of salmon treated by curcumin and piperine combined with vacuum packaging on day 0 **(A)**, day 4 **(B)**, and day 10 **(C)**; typical LF NMR T_2_ relaxation times **(D)** and MRI **(E)** of salmon treated by curcumin and piperine combined with vacuum packaging during cold chain logistics.

Figures 3A-C show the original NIR spectra of salmon filets different treatment groups in the early, middle, and late stages of cold chain logistics. These spectra had similar shape with the main features, in which several broad absorption maxima centered around 5,200 cm^–1^ (1,923 nm) and 7,000 cm^–1^ (1,428 nm). Absorption at these wavenumbers should be attributed to the first overtone of OH–NH stretching and a combination of OH stretching (64). The major differences among the spectra of the different treatments involved a baseline drift, which should be contributed to the different levels of moisture content. The reflectance of CP, VP, and VP+CP at typical wavenumbers of water were much lower than CK-TFT and CK-CON, indicating that they retained more moisture is the muscle, in accordance with the results of WHC. Similar drift pattern was also found in pork that the baseline drifted higher after the fresh pork meat suffered through freeze-thaw circles, due to the proteins relaxation and proteolysis (65).

### Low-field nuclear magnetic resonance

Figure 3D Shows the T_2_ transverse relaxation time of salmon under different treatment conditions during the cold chain logistics process, and shows that there are three peaks in the salmon meat, corresponding to three different moisture states (28). T_21_ is related to strongly bound water with the shortest relaxation time of 0.4-1.6 ms. T_22_ is related to trapped water, and its relaxation time is mainly concentrated in 49-55 ms, which is mainly trapped in the highly organized protein-dense myofibril network and the water in the tertiary and quaternary protein structures. T_23_ is related to free water outside of myofibrils, and its relaxation time is about 376-905 ms. The signal intensity of T_22_ in the control groups decreased rapidly in the later storage period, which might be mainly due to the destruction of the myofibril structure, leading to the transition from the trapped water to free water (T_23_). The appropriate cross-linking of amino acids and the prevention of myosin denaturation could be to account for the lower increase in water mobility (66). The myosin denaturation generated by the oxidative alterations might also affect the water mobility in protein molecules, as reported by Zhang et al. (67). It showed that the compound preservative combined with vacuum packaging has a certain effect on maintaining the moisture content of salmon samples, thereby delaying the spoilage of salmon.

Figure 3E Shows the MRI of salmon under different treatments in the cold chain logistics process, in which the different colors represent the moisture content. The redness means higher moisture content, and the blueness represents the low moisture content (68). With the increasing storage time, all groups showed a color change from red to blue, and the color of CK-TFT was significantly bluer and darker than the other groups, indicating that CK-TFT had the most severe water loss. It is demonstrated that temperature abuse might accelerate the protein degradation. VP+CP had higher yellowness and redness than other groups, indicating lower water loss. This result was also corresponding to the results of WHC, NIR and T_2_ relaxation time, indicating that VP+CP could maintain the water in the salmon sample more effectively, which meant lower degree of protein relaxation, degradation, and oxidation.

### Correlation analysis

**FIGURE 4:**
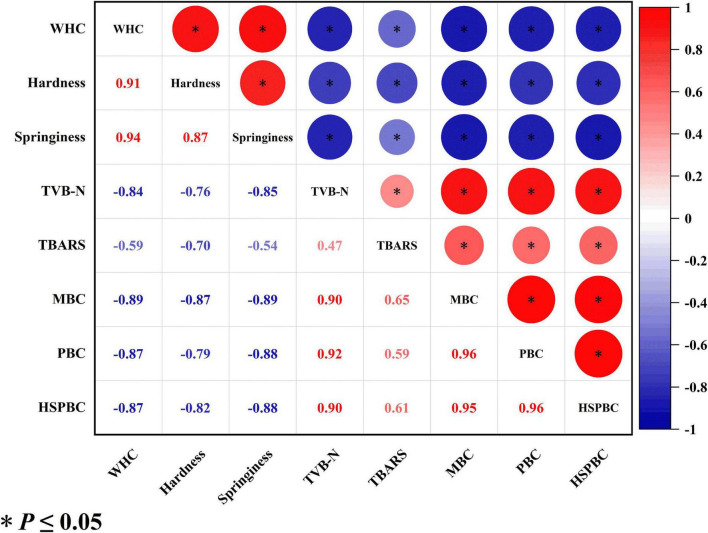
Correlation analysis of the quality indicators of salmon filets during cold chain logistics.

Figure 4 shows the correlation of various quality indicators of salmon filets. The positive/negative value of the data indicates that there is a positive or negative correlation between the indicators. The absolute value of each data represents the strength of the correlation between the two chemical indicators. The closer the data is to 1, the stronger the correlation between the two indicators. The correlation coefficients between TVB-N value and TVC, PBC and HSPBC were 0.9, 0.92 and 0.9, respectively, showing a strong positive correlation. The results indicated that bacterial metabolic activity could be the major reason for the increase of TVB-N. The correlation coefficients between the TVB-N value and WHC, hardness, and springiness were −0.84, −0.76, and −0.85, respectively. The correlation coefficients between WHC and TVC, PBC, and HSPBC were −0.89, −0.87, and −0.87, respectively, showing a strong negative correlation. WHC was strong positively corelated with hardness and springiness. To sum up, various quality indicators of salmon do not exist independently. As TVB-N is the major products of protein degradation, it is hypothesized that the degradation of protein process caused the loosening of muscle fibers, leading to the decrease of hardness, springiness, and WHC. Therefore, a technology which is capable of retarding the protein degradation and bacterial growth would be helpful to maintain the physiochemical quality of salmon filets.

## Conclusion

In this experiment, the effects of vacuum packaging, and the compound preservatives containing curcumin and piperine on the microbial growth, physiochemical indicators, texture properties, and water distribution of salmon during cold chain logistics suffered from temperature abuse were studied. The results showed that the hardness, springiness, and water holding capacity of different samples decreased to varying degrees, while the bacterial counts and TVB-N increased to a certain extent. The TBARS value and free amino acid contents first increased and then decreased during the whole period. Generally, the VP+CP had the highest levels of WHC, hardness, springiness, and lowest TBARS values. According to the result of NIR and LF-NMR analysis, the combined treatment VP+CP had higher moisture contents and lower moisture migration, in accordance with the changes of WHC, hardness, and springiness. The results highlighted that the temperature abuse was harmful to the quality of salmon, and VP+CP treatment could conquer the adverse effect of temperature abuse, and maintained the freshness of salmon most effectively, indicating that they had a synergistic effect. As spice recourse components, curcumin and piperine in combined with vacuum packaging could be a promising method for preservation of seafood.

## Data availability statement

The original contributions presented in this study are included in the article/supplementary material, further inquiries can be directed to the corresponding author/s.

## Author contributions

Y-FQ: conceptualization, funding acquisition, writing—review and editing. J-YY: writing—original draft preparation and data curation. Y-JY: writing—original draft preparation, data curation, and investigation. JX: supervision, and funding acquisition. S-PY: writing—review and editing and project administration. All authors have read and agreed to the published version of the manuscript.
